# A Mouse Model of Adoptive Immunotherapeutic Targeting of Autoimmune Arthritis Using Allo-Tolerogenic Dendritic Cells 

**DOI:** 10.1371/journal.pone.0077729

**Published:** 2013-10-24

**Authors:** Jie Yang, Yiming Yang, Yana Ren, Rufeng Xie, Hejian Zou, Huahua Fan

**Affiliations:** 1 Blood Engineering Laboratory, Shanghai Blood Center, Shanghai, China; 2 Division of Rheumatology, Huashan Hospital, Fudan University, Shanghai, China; Penn State University, United States of America

## Abstract

**Objective:**

Tolerogenic dendritic cells (tDCs) are immunosuppressive cells with potent tolerogenic ability and are promising immunotherapeutic tools for treating rheumatoid arthritis (RA). However, it is currently unknown whether allogeneic tDCs (allo-tDCs) induce tolerance in RA, and whether the numbers of adoptively transferred allo-tDCs, or the requirement for pulsing with relevant auto-antigens are important.

**Methods:**

tDCs were derived from bone marrow precursors of C57BL/B6 mice, which were induced *in* *vitro* by GM-CSF, IL-10 and TGF-β1. Collagen-induced arthritis (CIA) was modeled in D1 mice by immunization with type II collagen (CII) to test the therapeutic ability of allo-tDCs against CIA. Clinical and histopathologic scores, arthritic incidence, cytokine and anti-CII antibody secretion, and CD4^+^Th subsets were analyzed.

**Results:**

tDCs were characterized *in* *vitro* by a stable immature phonotype and a potent immunosuppressive ability. Following adoptive transfer of low doses (5×10^5^) of CII-loaded allo-tDCs, a remarkable anti-arthritic activity, improved clinical scores and histological end-points were found. Serological levels of inflammatory cytokines and anti-CII antibodies were also significantly lower in CIA mice treated with CII-pulsed allo-tDCs as compared with allo-tDCs. Moreover, treatment with allo-tDCs altered the proportion of Treg/Th17 cells.

**Conclusion:**

These findings suggested that allo-tDCs, especially following antigen loading, reduced the severity of CIA in a dose-dependent manner. The dampening of CIA was associated with modulated cytokine secretion, Treg/Th17 polarization and inhibition of anti-CII secretion. This study highlights the potential therapeutic utility of allo-tDCs in autoimmune arthritis and should facilitate the future design of allo-tDC immunotherapeutic strategies against RA.

## Introduction

Rheumatoid arthritis (RA) is an autoimmune and chronic inflammatory disorder that follows a heterogeneous course. RA is characterized by persistent joint inflammation that results in progressive destruction of cartilage and the underlying bone. The inflammatory process in RA involves symmetrical and often bilateral swelling of the joints that reflects hyperplasia of the synovial membrane and a cellular infiltration by monocytes, macrophages, T and B cells, mast cells and dentritic cells (DCs) [1]. Current approaches for treating RA use immunosuppressive drugs and biological agents, which might induce generalized immune suppression and an increased risk of opportunistic infections [2]. Thus, new therapeutic approaches should be aimed at dampening inflammation and promoting tolerance toward arthritic antigens without compromising protective host immunity [3].

The most potent antigen-presenting cells (APCs) are DCs, which are regarded as the master regulators of host immunity, including alloreactive immune responses. It has been previously reported that DCs exposed to immunosuppressive cytokines like IL-10 and TGF-β, could potentially induce tolerance [4]. This specific subset of DCs is termed ‘tolerogenic DCs’ (tDCs), which typically present low levels of self-peptide-MHC complexes (signal 1) coupled with limited cell-surface co-stimulatory molecule expression (signal 2) and secretion of pro-inflammatory cytokines (signal 3). The net result of this process leads to T cell anergy and apoptosis. Thus, tDCs not only reflect an incomplete or altered status in DC differentiation, but they are also considered as potential immunotherapeutic tools in the management of autoimmune conditions like RA [5]. 

Based on the distinct functional properties of tDCs in promoting tolerance, previous proof-of-concept animal models of RA showed that established arthritis could be significantly suppressed following treatment with autologous DC [6-9]. However, the potential in using allo-tDCs has many advantages. This includes the ability to propagate DC in advance and in relatively large numbers, the ability to screen DC for functional bio-activity, and to screen DC against pathogen contamination and the deployment of a range of quality control measures consistent with both good laboratory and good manufacturing practices. Thus, if allo-tDCs can be shown to ameliorate RA, these cells could be employed as a novel immunotherapeutic tool for targeting RA. However, it remains poorly understood whether allo-tDCs exert similar suppressive activity as compared recipient-type tDCs in RA. Additionally, it is unclear whether allo-tDCs require pulsing with relevant autoreactive antigens, or the optimal number of allo-tDCs that should be administered. 

CIA is a well established mouse model of human RA [10]. This model is frequently used to develop and test novel therapies. Like RA, CIA is characterized by severe inflammation and cellular infiltration of the synovial tissues, which leads to cartilage and bone destruction. Moreover, T cells play a pivotal role in the pathogenesis of both RA and CIA. In this study, the ability of mouse BM-derived allo-tDCs propagated in IL-10 and TGF-β1 to function as an adoptively transferred immunotherapy in CIA was investigated. Our data suggest that allo-tDCs, and especially those pulsed with relevant autoreactive antigens, effectively reduced the severity and progression of CIA in a dose-dependent manner. Moreover, this reduction was associated with prolonged inhibition of anti-CII antibody secretion, modulation of inflammatory cytokine secretion and Th17/Treg polarization. Our findings indicated the potential immunotherapeutic utility of allo-tDCs in treating autoimmune arthritis.

## Results

### Exposure to IL-10 and TGFβ1 altered the surface phenotype, expression of cytokines and function-associated molecules in BM-derived myeloid DC

**Figure 1:**
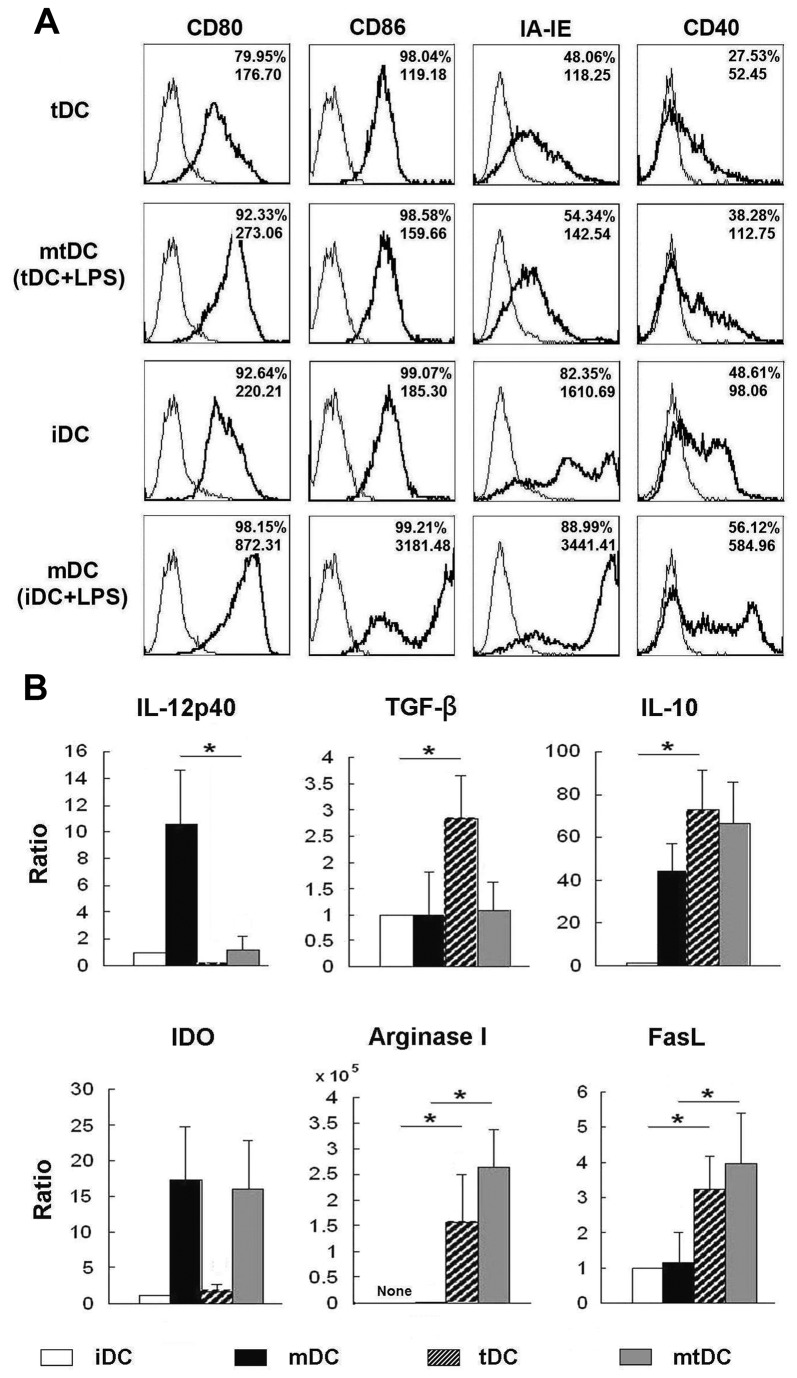
Characteristic profile of tDCs derived from D1 mice. iDCs, mDCs, tDCs and mtDCs were induced in vitro as described in the Materials and Methods section, following which CD11c+ cells were harvested. (**A**) These four types of DC were stained with IA-IE, CD80, CD86, CD40 (thick lines) respectively or isotype-matched mAbs (thin lines) and the expression of those markers was analyzed by FACS flow cytometry. The frequency of positively stained cells and MFI of a representative of 10 independent experiments are shown in the FACS profile. (**B**) Expression of IL-12p40, IL-10, TGF-β, FasL, IDO and arginase by DC were determined by real-time PCR. All results were normalized to the expression of the housekeeping gene β-actin, and expressed as the mean ± SD of six independent experiments. *P <0.05 in comparisons of iDCs vs. tDCs, or mDCs vs. mtDCs by unpaired t-tests.

Initial experiments investigated that tDCs derived from mouse BM were induced by GM-CSF with additional IL-10 and TGF-β1. These were conditions that did not affect DC development from replicating BM progenitors [11]. In the present study, a large proportion of induced BM cells were positive for CD11c (>80%), which indicated that these cells were of a DC phenotype (Supplement 1). The lower expression of MHC II molecules (IA-IE) and CD40 was detected on tDCs as compared with immature DCs (iDCs) (Figure 1A). Following stimulation with LPS for 48 h, the incidence of cells expressing IA-IE, CD80, CD86 and CD40 and the mean fluorescence intensity (MFI) for each of these molecules was consistently lower on mature tDCs as compared mature DCs (mDCs).

Additionally, cytokine production and the expression of function-associated molecules by tDCs were assessed. We found that tDCs displayed higher levels of IL-10, TGF-β, Fas Ligand (FasL) and arginase as compared with iDCs. In particular, enhanced levels of FasL, indoleamine 2, 3-dioxygenase (IDO) and arginase were detected after stimulation of tDCs with LPS. However, IL-12p40 production by tDCs was negligible, even upon stimulation by LPS (Figure 1B). Taken together, these data indicated that tDCs induced by GM-CSF with IL-10 and TGF-β1 maintained a tolerogenic surface phenotype even after LPS stimulation, and expressed substantial levels of ‘immunosuppressive’ function-associated molecules. 

### CII-pulsed allo-mtDCs exhibited potent suppressive activity against CD4+T cell proliferation in vitro

To demonstrate the ability of allo-tDCs to suppress the proliferation of effector T cells, we used a proliferation assay as a functional readout. In these proliferation assays, CD4^+^ T cells (the responders) were isolated from CIA mice, and CII-loaded mDCs derived from normal D1 mice, were used as stimulators. In the absence of suppressors, the responder cells underwent vigorous proliferation, generating a large numbers of dividing T cells in culture. However, this intense proliferation was suppressed by the presence of B6 mouse-derived allo-tDCs (suppressors), when cultured at different suppressor-to-responder cell (S: R) ratios.

**Figure 2:**
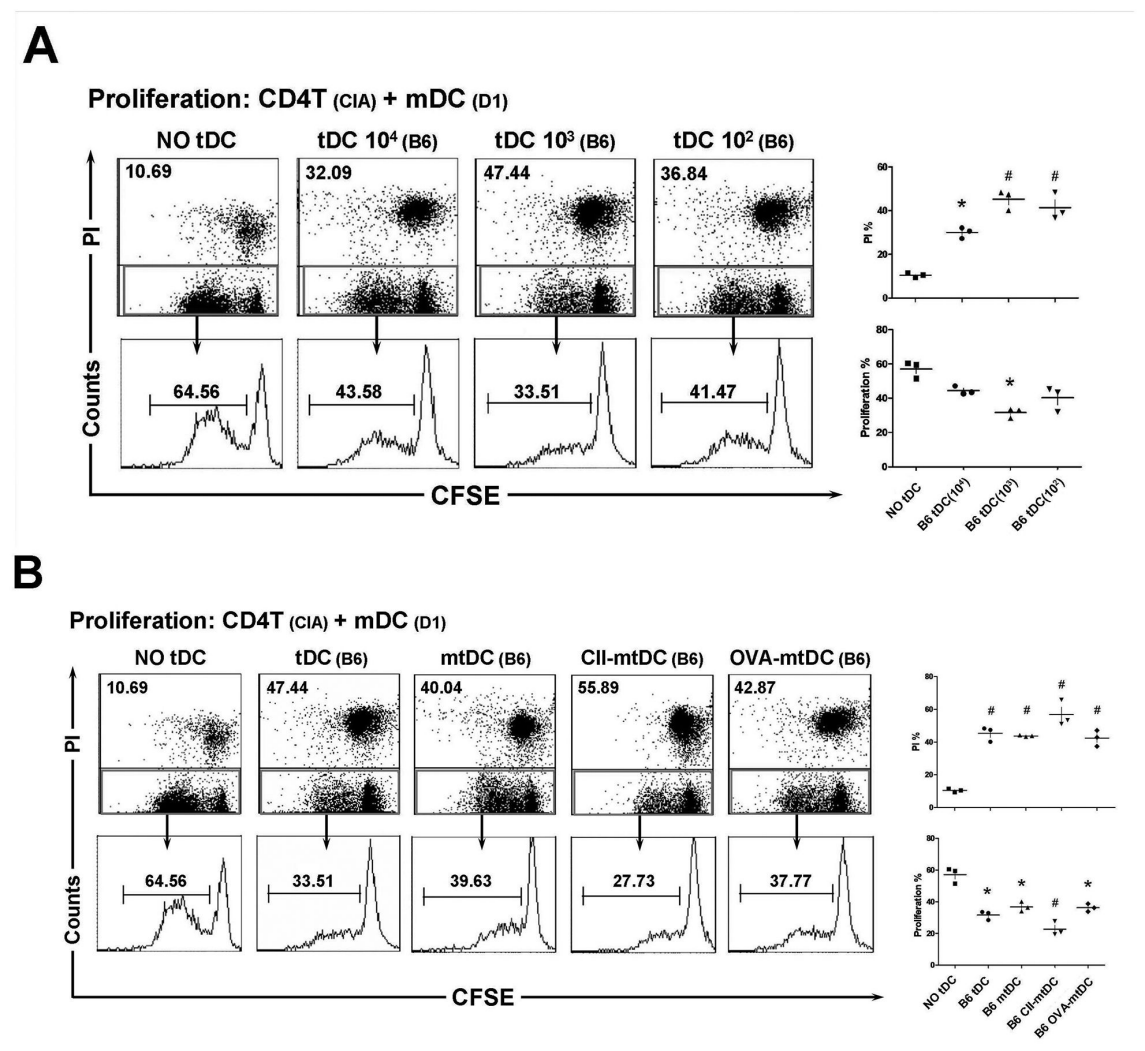
Allo-tDCs exhibited potent immunosuppressive activity in antigen-specific CD4^+^T cell proliferation when at a suitable S: R ratio in vitro. In the proliferation assays, CD4^+^T cell (responders) were isolated from CIA mice (H-2Kq) SC, which were then stained with CFSE, and stimulated with CII-loaded mDCs (stimulators) derived from bone marrow precursors of normal D1 mice. (**A**) Different doses of allo-tDCs (suppressors) from B6 mice were added to the proliferation assay, at a suppressor: responder (S:R) ration of 1:10, 1:100 and 1:1000. (**B**) Different sub-types of modified allo-tDCs were added to the proliferation assay, at an S:R ratio of 1:100. After co-culture for 4 d, cells were harvested and analyzed by FACS. Progressive dilution of CFSE and the rate of PI positive cells were used as measures of proliferation and apoptotic death of responder cells respectively. The results are representative of three independent experiments. *P <0.05 and # *P* <0.01, as compared with No-tDCs group by unpaired *t*-tests.

At all of the responder-to-tDC ratios, allo-tDCs promoted both apoptotic cell death and suppressed the proliferation of CIA-CD4^+^T cells (Figure 2A). Interestingly, the low dose of allo-tDCs, (10^3^ cells and an S: R ratio of 1:100) resulted in the most effective inhibition of cellular expansion and death of CD4^+^ T cells. However, higher or lower doses of allo-tDCs resulted in weaker suppression and dampened apoptosis of CD4^+^ T cells. Furthermore, proliferation of CD4^+^ T or CD8^+^ T cells was negligible following stimulation with allo-tDCs alone (Supplement 2). These results indicated that allo-tDCs retained a highly effective inhibitory potency and exerted suppressive activities in a dose-dependent manner *in vitro*.

Next, we tested whether CII-pulsed allo-mtDCs induced tolerance to CIA-CD4^+^ T cell-dependent proliferation in an antigen-specific manner. This was assessed by the proliferation assay as described above, in which the S: R ratio was set at 1:100 (Figure 2B). CII-pulsed allo-mtDCs showed more effective inhibition of antigen-specific CD4^+^ T cell expansion, and induced more intense death of responder cells as compared with either allo-tDCs or allo-mtDCs. Additionally, there were insignificant differences between the suppression of T cell proliferation seen with either allo-mtDCs or OVA-pulsed allo-mtDCs (*P*>0.05). Together, these data showed that CII loaded allo-mtDCs potently suppressed antigen-specific CD4^+^ T cell proliferation and enhanced CD4^+^ T cell death at appropriate S: R ratios. 

### Therapeutically optimal doses of allo-tDCs displayed effective anti-arthritic activity in CIA mice

**Figure 3:**
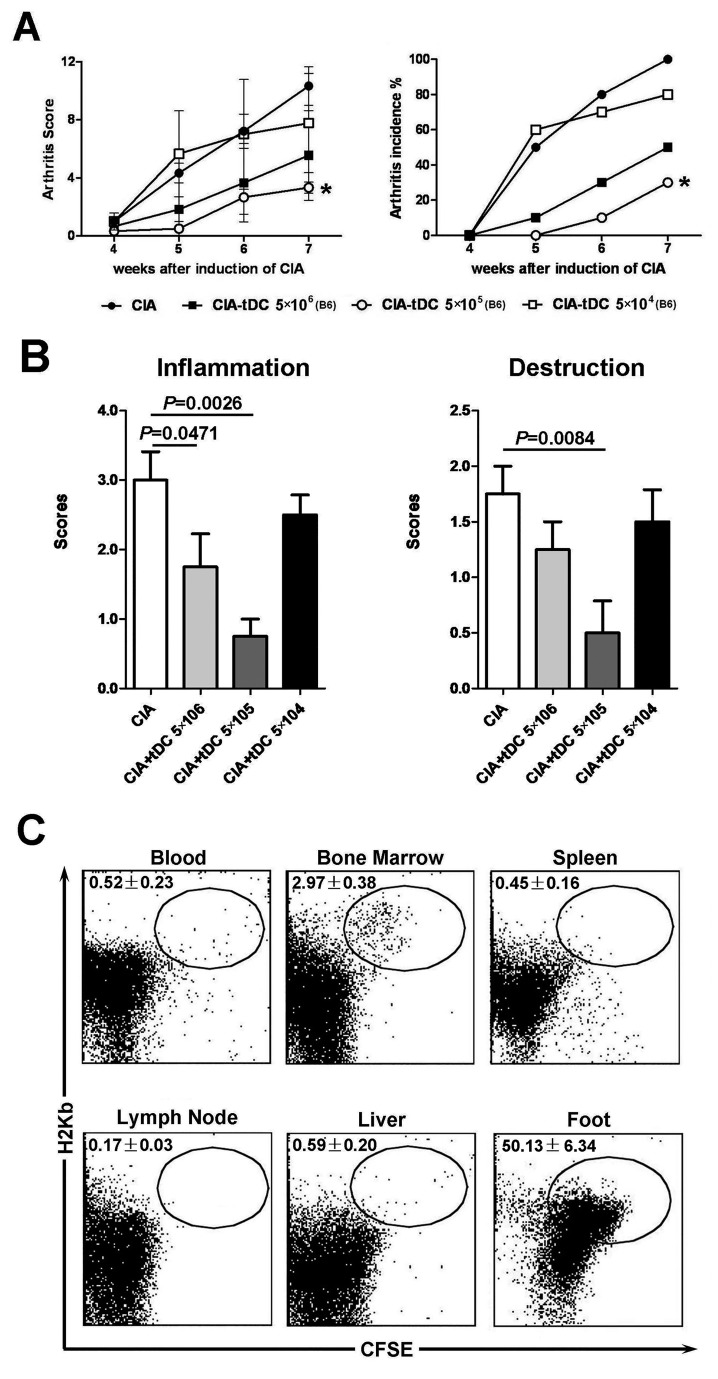
Therapeutically optimal doses of allo-tDCs promoted an effective anti-arthritic activity in CIA mice. Three different doses of allo-tDCs (ranging from 5×10^6^ to 5×10^4^ cells) were adoptively transferred following the onset of experimentally induced CIA. Mice were scored for clinical signs of arthritis in the limb joints by macroscopic examination three times a week. Limb joint arthritis was assessed by an established scoring system. (**A**) Arthritic score and incidence following adoptive transfer of different doses of allo-tDC in each group (n=10) during the observation period are shown. *P <0.05 as compared with CIA mice by unpaired *t*-tests. (**B**) Hind paw specimens from recipient mice treated with or without the different doses of allo-tDCs were collected at week 3 following onset of CIA. The histopathologic scores of inflammation and destruction are expressed as the mean ± SEM of five individual experiments. (**C**) 5×10^5^ allo-tDCs labeled CFSE were transferred into the CIA mice. After 7 days, mononuclear cells from the blood, BM, spleen, liver, lymph node and feet were isolated and stained H-2K^b^. And H-2K^b+^CFSE^+^ cells were determined as allo-tDCs derived from B6 mice by flow cytometry. Data are representative of three independent experiments.

The CIA model of arthritis is a well-established method for the evaluation of therapeutic interventions in autoimmune arthritis. Therapeutically optimal doses of allo-tDCs were transferred into CIA-induced mice, following which the arthritic index and histopathology were examined. In these experiments, three different doses of allo-tDCs (that ranged from 5×10^6^ to 5×10^4^) were injected into CIA mice when onset of CIA was confirmed. Repeated experiments demonstrated remarkable anti-arthritic activity and an improved clinical score in CIA mice, following adoptive transfer of low doses of allo-tDCs. Arthritic symptoms were not observed in CIA mice that were adoptively transferred with 5×10^5^ allo-tDCs, whereas lower doses (5×10^4^/animal) or higher doses (5×10^6^/animal) were ineffective in reducing the severity of arthritis. During the 3 wk observation period, mice that were vaccinated with 5×10^5^ allo-tDCs after CIA-induction demonstrated the lowest arthritis scores as compared mice that were vaccinated with other doses or the untreated control CIA mice (Figure 3A). 

Next, histological differences in CIA in mice treated with or without varying doses were assessed on the 21st day following the onset of arthritis (Figure 3B). Examination of the histopathological specimens revealed that the joints of mice treated with 5×10^5^ allo-tDCs showed the most remarkable decrease in destruction of the cartilage and inflammatory cell infiltration as compared with control CIA mice. Furthermore, remarkable levels of allo-tDCs (50.13 ± 6.34%, n=3) derived from B6 mice (H2-Kb^+^CFSE^+^) were detected in the feet of CIA mice on Day 7 after adoptive transfer. Further, low levels of allo-tDCs (2.97 ± 0.38%, n=3) were also detected in the BM, but few allo-tDCs survived in the blood, spleen, lymph node and liver (Figure 3C, negative control shown in Supplement 3). These results were consistent with those made from the *in vitro* experiments and suggested that allo-tDCs could survive in CIA mice, and when adoptively transferred at optimal doses, could potently inhibit the progression of RA.

### Inhibition of the development of collagen-induced arthritis by CII-pulsed allo-mtDCs

**Figure 4:**
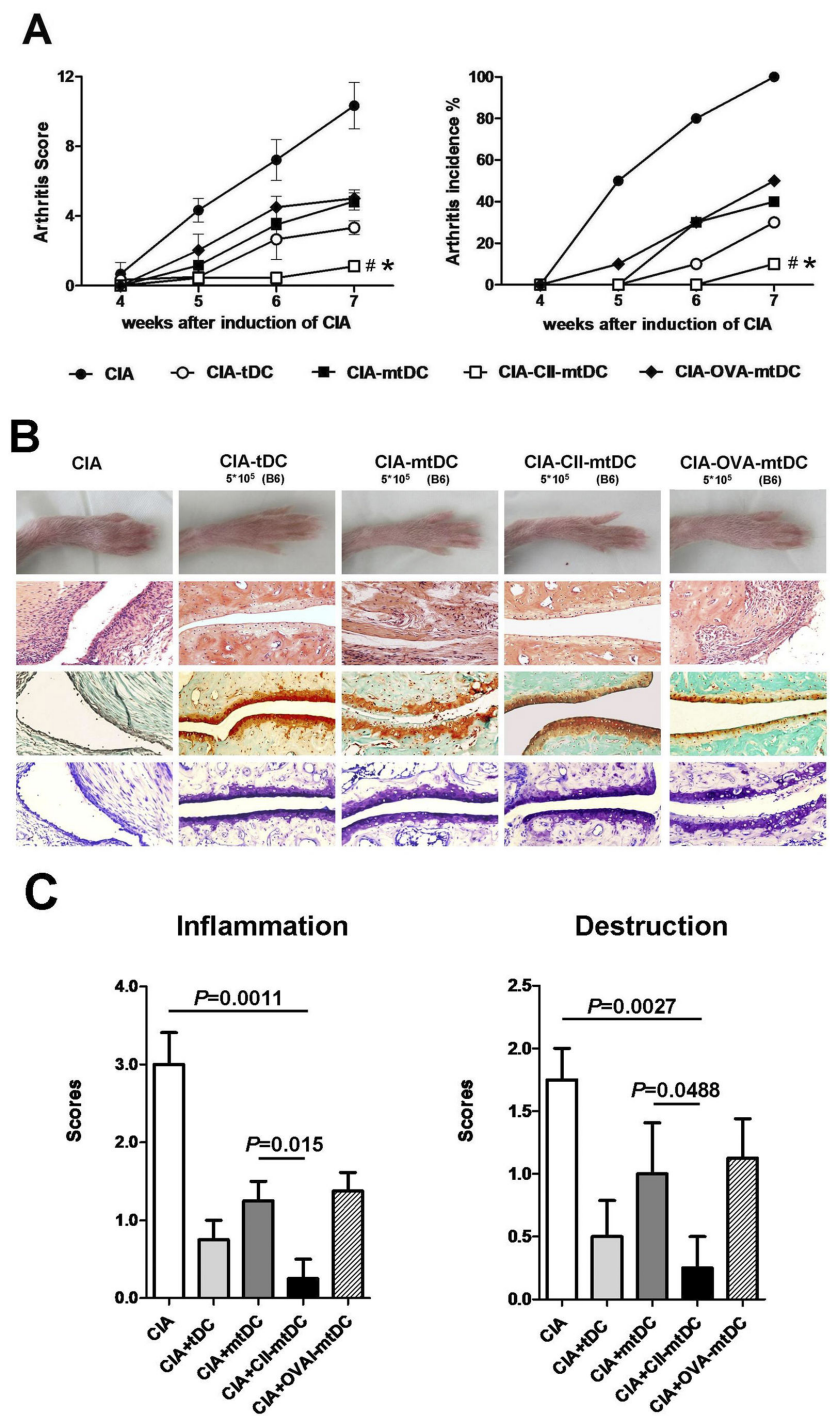
Different subsets of modified allo-tDCs inhibited development of CIA. Allo-tDCs were stimulated by LPS and pulsed with either CII peptide, OVA (as an irrelevant antigen), or the DC were untreated, and adoptively transferred into CIA mice at the same density (5×10^5^/animal). (**A**) Each group of mice (n=10) was scored for clinical signs of arthritis in the limb joints by macroscopic examination three times a week. Limb joint arthritis was scored using an established scoring system. Arthritic scores and incidence following adoptive transfer of different densities of allo-tDC during the observation period are shown. *P <0.05 as compared with mice treated with allo-mtDCs by unpaired *t*-tests and #*P* <0.01 as compared with CIA mice by unpaired *t*-tests. (**B**) Hind paws were collected at week 3 following onset of CIA. Photographs of the affected paws were taken (top) and tissue was stained with H&E (upper middle, showing synovial joint inflammation), Safranin O (lower middle, showing cartilage erosion) or toluidine blue (bottom panel, showing cartilage erosion). (**C**) It showed histopathologic scores of inflammation and destruction, which are expressed as the mean ± SEM of five independent experiments.

As the above observations suggested, significant decreases in arthritis severity seen in CIA mice could be achieved by transferring 5×10^5^ allo-tDCs. We then wished to determine whether this effect was antigen-specific. Allo-tDCs were stimulated by LPS and pulsed with either CII peptide, OVA (as an irrelevant antigen), or left untreated. Allo-tDCs were then adoptively transferred into CIA mice at the same doses (5×10^5^/animal). We found that both mtDCs and OVA-pulsed mtDCs induced a similar effect as that seen for tDCs (Figure 4). Also, when compared with the therapeutic effects of allo-tDCs and allo-mtDCs, treatment with the same doses of CII-pulsed allo-mtDCs more clearly inhibited the development of CIA, which was substantiated by both clinical scores and in the incidence of arthritis (Figure 4A). Similar results also showed an anti-arthritic effect of autologous tDCs by an antigen-specific mode of action (Supplement 4A-B). Additionally, we found that the histological examinations were consistent with the clinical scores (Figure 4B-C). These observations confirmed that treatment of CIA with CII-pulsed allo-mtDCs was the most effective, and suggested that the beneficial effects of CII-pulsed and LPS-stimulated allo-tDCs were antigen-specific.

### Allo-tDC-mediated inhibition of CIA was associated with modulated cytokine secretion, prolonged inhibition of anti-CII antibodies, and polarization of Treg/Th17 balance

**Figure 5:**
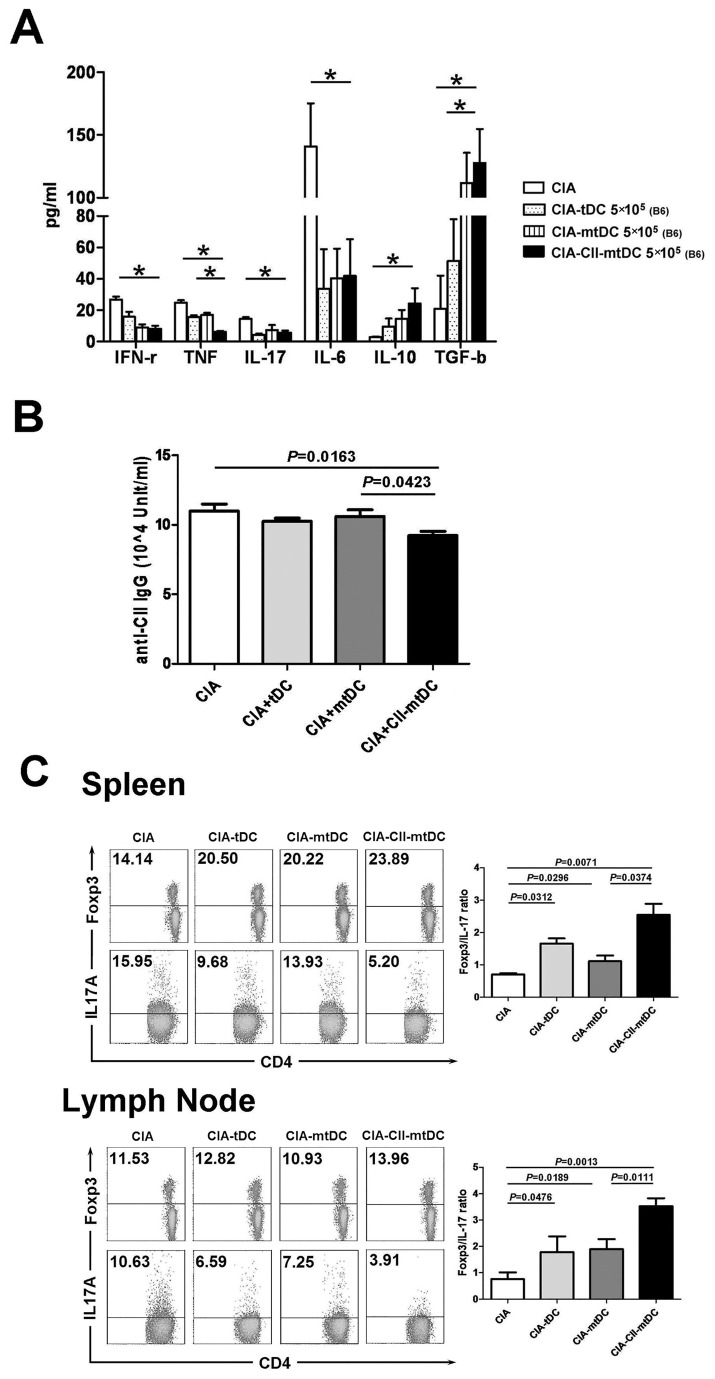
Inhibition of CIA is associated with modulation of cytokine secretion, prolonged inhibition of anti-CII antibody secretion, and polarization of the Treg/Th17 balance. (**A**) Serum was collected from each group of mice that were treated with differentially modified allo-DCs at 3 weeks following the onset of CIA. Serum levels of IFNγ, TNFα, IL-6, IL-10, and IL-17 were measured by CBA assays and TGFβ secretion was measured by ELISA. Data are reported as mean ± SEM. *P <0.01 as compared with the indicated groups by unpaired *t*-tests. The results of five replicated independent experiments were pooled. (**B**) Serum levels of total CII-specific immunoglobulin in each group of mice treated with differentially-modified allo-DCs, 3 weeks after the onset of CIA was determined by ELISA. Results of five replicated independent experiments were pooled. (**C**) CD4^+^T cells from spleen and the inguinal lymph nodes from CIA mice that had been treated with or without subsets of modified allo-tDCs at the third week after the onset of arthritis were collected and stained intracellularly with anti-Foxp3 mAb and anti-IL-17 mAb. FACS flow cytometry was used to measure the percent frequency of positively stained cells, and the frequency of Treg/Th17 cells was expressed as the mean ± SEM of five independent experiments.

To study the *in vivo* effect of allo-tDC treatment on CIA, the secretion of IFN-γ, TNF-α, IL-6, IL-10, IL-17 and TGF-β in the serum of CIA mice treated with or without modified allo-tDCs was analyzed 21 days after the first tDC injection. The levels of IFN-γ, TNF-α, IL-17, and IL-6 were significantly lower, and the levels of IL-10 and TGF-β were higher in CIA mice that had been adoptively transferred with allo-CII-mtDCs as compared those mice treated with allo-tDCs or allo-mtDCs (Figure 5A). Clearly, the attenuation of CIA in mice following adoptive transfer of allo-tDCs was a consequence of their immune-modulating effect on the secretion of various cytokines.

The importance of antibodies in the development of CIA pathology is also well described [12]. Serum levels of CII-specific immunoglobulin in allo-CII-tDC-treated mice were markedly lower by 21 days following the onset of arthritis as compared with CIA mice (Figure 5B). Additionally, treatment with either allo-tDC or allo-mtDC failed to significantly reduce the secretion of CII-specific IgG. This mode of therapy also did not significantly differ between those mice treated with/without allo-tDCs or allo-mtDCs. This observation suggested that CII-pulsed allo-mtDCs inhibited CII-specific antibody responses.

Furthermore, the ratio of Treg/Th17 was considered an important indicator of the severity of CIA. Thus, we determined the percent frequency of Th17 (CD4^+^IL-17^+^) and Treg (CD4^+^Foxp3^+^) cells in CD4^+^ T cells isolated from the spleen or the inguinal lymph nodes of CIA mice that were treated with or without varying modified allo-tDCs on day 21 following the onset of arthritis (Figure 5C). We found that treatment with allo-tDCs altered the proportion of Treg/Th17 cell populations, irrespective of whether allo-tDCs were stimulated with LPS, pulsed with CII or in the complete absence of such stimulation. However, an increase in the number of Tregs and a decrease in the percent frequency of Th17 cells were seen following treatment with allo-tDCs, which resulted in an increase in the Foxp3 versus IL-17 ratio. The highest Foxp3/IL-17 ratio was seen in CIA mice that were treated with CII pulsed allo-mtDC as compared treatment with either allo-tDC or allo-mtDC.

Taken together, these findings demonstrated that allo-tDCs, and especially following their stimulation by LPS and pulsing with relevant antigens, could effectively dampen the severity and progression of CIA. This reduction in CIA disease activity was associated with modulation of cytokine secretion, prolonged inhibition of anti-CII antibodies, and polarization of the Treg/Th17 balance. 

## Discussion

Over the past decade, detailed studies on both mouse and human tDCs, and their relation to the regulation of allo-immunity have indicated the importance of tDCs as modulators of autoreactive T cell responses [1]. Many studies have demonstrated the potential of DC in the treatment of autoimmune diseases [8,9,13-15]. However, the use of allo-tDC-based immunization as a strategy to induce tolerance remains to be elucidated. In this study, we evaluated the tolerogenic capacity of allo-tDCs, which were propagated from mouse BM in the presence of GM-CSF, IL-10 and TGF-β1. We studied the effect of allo-tDCs in modulating autoreactive T cell responses both *in vitro* and *in vivo*. The suppressive function of allo-tDCs was observed in proliferation assays and in an established CIA mouse model of arthritis. Our results indicated that allo-tDCs might be applicable in the treatment of RA.

Phenotypically, tDCs propagated from BM cells with IL-10 and TGF-β1 express lower levels of MHC II and co-stimulatory molecules as compared with iDCs. This observation is consistent with other studies [16,17]. In terms of cytokine secretion, tDCs produced minimal levels of IL-12 and expressed comparably higher levels of both IL-10 and TGF-β. Even after stimulation by LPS for 48h, tDCs were comparatively resistant to maturation, a characteristic that might be involved in their reduced ability to function as APCs. The low APC capacity of tDCs was demonstrated in both allogeneic and autologous memory responses against soluble antigen, which suggested their potential in a variety of conditions [4]. Based on the distinct characters of tDCs in tolerance, we have reason to believe the possibilities of allo-tDCs in the immune modulation of autoreactive responses. Furthermore, tDCs maintained the tolerogenic surface phenotype and expressed FasL, IDO and arginase. These are markers that suggest an important role in driving allo-CD4^+^T cell apoptosis. Thus, a variety of apoptosis-inducing factors might render allo-tDCs, even at low doses, highly effective in the suppression of T cell proliferation by a mechanism dependent on the induction of apoptosis.

Noticeably, though more than 80% of bone marrow cells were CD11c^+^ cells after polarization, there were still fewer than 20% of other cell subsets. Moreover, BM is known as a reservoir for Treg cells, which could inhibit antigen-induced arthritis, and there is a large population of myeloid-derived suppressor cells (MDSCs) with suppressive activity in the BM. Thus, we determined what cellular subsets of CD11c^-^ cells were present by FACS analysis. As shown in supplement 1, the Foxp3^+^ cells (0.36 ± 0.27% in CD11c^-^ cells) were barely detected, suggesting there were few Tregs in contamination. The percentage of CD11b^+^Gr1^+^ defined MDSCs [18] were 46.97 ± 5.13% in the CD11c^-^ cell sub-population and about 5% of the total cells. Previous studies clearly showed that MDSCs have the ability to suppress T cell responses and could inhibit autoimmune diseases, such as EAE, SLE, IBD, and type I diabetes in mouse models [19]. Additionally, Jiao et al. found that increased circulating MDSCs correlated negatively with Th17 cells in patients with rheumatoid arthritis [20]. Therefore, we found a firm rationale to speculate that MDSCs could inhibit CIA. In addition, there were low levels of CD11b^+^Gr1^-^ and CD11b^-^Gr1^+^ cells (2.86 ± 0.14 and 1.61 ± 0.77 in total cells), whose functions on CIA could be ignored. We also isolated CD11c^+^tDCs after *in vitro* polarization by immunomagnetic bead isolation. After transferring the same doses of cells (5×10^5^) cultured *in vitro*, there were no significant differences seen between the isolated-CD11c^+^tDCs-treated group and the non-isolated-tDCs (containing a few MDSCs at about 2-3×10^4^)-treated group (data not shown). So, considering the notion that it is highly unlikely that MDSC could also inhibit CIA and the number of MDSCs is extremely limited, we did not isolate tDCs in the subsequent study. 

As is currently known, CIA has traditionally been performed in MHC classII A^q^-expressing mice, such as D1 mice. But special attention should be paid to the knowledge that chicken CII could induce arthritis in B6 mice, whose background expresses the A^b^ haplotype [21]. Moreover, the disease in B6 mice is milder, but more chronic, with more pronounced and more persistent T-cell responses [22,23]. This is different with D1 mice, in which bovine, mouse, chicken and rat CII all reduced CIA. In Inglis’ study, the strong proliferative and cytokine responses to relevant CII in the lymph node cell cultures from B6 mice were detected [23]. 

To some extent, these data suggested that tDCs from B6 mice might have the ability to present chicken CII peptide, not mouse or bovine CII. But the reason why only chicken CII could induce arthritis in B6 mice remains unknown. It was presumably due to the differences in the amino acid sequence between chicken and any other species in which CII was established, and H-2Kb class II molecules could recognize/present this type of peptide. So, in our present study, the *in vitro* and *in vivo* data showed that pulsing chicken CII peptide could help the tDCs generate an antigen-specific suppressive effect. 

Interestingly, the numbers of adoptively transferred allo-tDCs determined the fate of subsequent immune reactions. Data from other studies indicated that optimal treatment regimens vary according to the type of tolerogenic DC [14,24]. A previous study also showed that a dose of 2.5×10^6^ TNF-treated DCs was pathogenic [13]. Other studies also confirmed that there were no adverse effects associated with the adoptive transfer of higher doses of dexamethasone/vitamin D3-treated tDCs, which indicated that this type of tDC were safe even at higher doses [25]. 

In our repeated experiments, low-doses (5×10^5^/mice) of allo-tDCs that were induced by IL-10 and TGF-β exerted a more potent suppressive activity in CIA mice. Symptoms of CIA in the mice treated with 5×10^6^ allo-tDCs were comparatively accelerated, although higher doses were found to transiently inhibit CIA in the early phase of established CIA. Furthermore, treatment with lower doses (5×10^4^/mice) of allo-tDCs was insufficient to effectively suppress the progression of arthritis. These observations were further supported by immunological analyses *in vitro*. 

Several studies have shown that multiple mechanisms are involved in the inhibitory abilities of auto-tDCs *in vivo* [26-28]. Our study has confirmed that allo-tDCs played a suppressive role *in vivo* by similar mechanisms, including modulation of cytokine secretion, prolonged inhibition of anti-CII antibodies and polarization of the Treg/Th17 balance. Following treatment with allo-tDCs, the serological levels of IFN-γ, TNF-α, IL-17 and IL-6 were reduced. By contrast, the levels of both IL-10 and TGF-β were enhanced in CIA mice. In this regard, we suggest that a tolerant state was established with allo-tDC treatment *in vivo*, which was apt to promote Treg cell differentiation and proliferation, while limiting the differentiation of Th17 cells *in vivo*. As expected, it was found that there were increases in the number of Foxp3^+^CD4^+^T cells and decreases in the percent frequency of IL-17^+^CD4^+^ T cells in SC and LN. By contrast, it has been demonstrated that Tregs could suppress the maturation of DCs by selectively down-regulating CD80/CD86 [29]. Thus, allo-tDCs could maintain their tolerogenic phenotype and function, thus promoting a potent tolerogenic microenvironment – a feature that was demonstrated by reduced presentation of CIA symptoms and inhibition of CIA progression.

Clearly, optimal doses of allo-tDCs were effective at treating arthritis following their adoptive transfer into CIA mice. When CII-pulsed allo-tDCs were used, their therapeutic effect on CIA was markedly enhanced during the observation period (3 weeks after treatments). This suggested that the targeting of type II collagen-specific T cells by allo-tDCs was important for their therapeutic effect. This was consistent with other studies using dexamethasone/vitamin D3-treated tDCs [25]. However, 3 wk after allo-CII-mtDCs transfer, the severity and development of CIA was not as effectively blocked (data not shown). This implied that the efficacy of a single cellular therapy was limited and it was necessary to modulate the transfer frequency. This could mediate a durable inhibition of CIA.

In conclusion, our study has shown that allo-tDCs could effectively dampen the severity and progression of CIA in a dose-dependent manner and especially following priming of those DC with relevant autoreactive antigens. This suppression of CIA was associated with modulation of inflammatory cytokine secretion, prolonged Th17/Treg polarization, and inhibition of anti-CII antibody secretion. Our findings suggest the potential therapeutic use of allo-tDCs for treating autoimmune arthritis and in facilitating the design of future immunotherapeutic trials in patients with RA.

## Materials and Methods

### Mice

Wild-type male C57BL/6 (B6, H-2K^b^) and DBA/1J (D1, H-2K^q^) mice (aged 8 wk) were obtained from the Shanghai Laboratory Animal Center of the Chinese Academy of Science (SLACCAS, China). Animals were housed in a specific pathogen-free environment with free access to drinking water that was supplemented with gentamicin sulfate. Experiments were conducted according to the guidelines of the Institutional Animal Care and Use Committee of the Chinese Association for Laboratory Animal Sciences.

### Ethics Statement

This study was carried out in strict accordance with the recommendations in the guidelines of the Institutional Animal Care and Use Committee of the Chinese Association for Laboratory Animal Sciences. The protocol was approved by the Committee on the Ethics of Animal Experiments of Shanghai Blood Center (Permit Number: 11-0002). All surgery was performed under diethyl ether, and all efforts were made to minimize suffering. 

### Induction and Evaluation of CIA

CIA was induced in male D1 mice by subcutaneous injection with 50 ug Chicken CII protein (Chondrex, Redmond, WA, USA) emulsified with an equal volume of complete Freund’s adjuvant (Difco, Detroit, MI, USA). On day 21, mice received a booster injection with 50 ug of CII in incomplete Freund’s adjuvant (Difco). After approximately 7 days, the onset of CIA was confirmed.

Beginning 4 wk after immunization, mice were scored three times per week for clinical evidence of arthritis of the limb joints by macroscopic examination. Limb joint arthritis was scored using an established scoring system as follows: no detectable arthritis, 0; erythema and mild swelling, 1; mild erythema and mild swelling involving the entire paw, 2; severe swelling and redness from the ankle to digits, 3; and maximal swelling and redness or obvious joint destruction associated with visible joint deformity or ankylosis, 4. The clinical scores for each mouse were the sum of the scores of the four limbs, and the maximal score for each mouse was sixteen. Two independent observers, without knowledge of the experimental protocol, performed the scoring. 

### Preparation of DCs

DCs were prepared as described [30] with some modifications. In brief, B6-derived BM cells were seeded at a density of 5×10^5^ cells/ml in RPMI 1640 medium supplemented with 10% fetal bovine serum (Invitrogen). On day 0, recombinant murine GM-CSF (20 ng/ml, PeproTech) was added to the cultures, following which, the cultures were pulsed with fresh medium, GM-CSF, recombinant murine IL-10 (15 ng/ml, PeproTech), and recombinant human TGF-β1 (15 ng/ml, PeproTech) on days 4 and 7. After 10 d in culture, non-adherent CD11c^+^ cells were collected as the tolerogenic subset of DCs (tDCs). Mature tDCs (mtDCs) were harvested after stimulation with lipopolysaccharide (LPS, 100 ng/ml, Sigma) for the final 48 h of culture, and CII-mtDCs were those that were pulsed with CII during LPS-mediated maturation. Immature DCs (iDCs) were propagated under the same conditions in GM-CSF alone and mature DCs (mDCs) were generated following exposure to LPS for 48 h.

### Flow cytometric analysis

DCs were stained with anti-mouse FITC-CD11c, PE-CD80, PE-CD86, PE-IA-IE, and PE-CD40 for phenotypic analysis. Anti-mouse mAbs were obtained from BD Biosciences/Pharmingen. The frequency of percent positive cells and MFI were analyzed by CellQuest software (Becton-Dickinson, Germany) by comparison with cells stained with PE-labeled isotype control mAbs.

### Quantification of cytokines and function-associated molecules

Real-time quantitative PCR was performed on an ABI Prism 7500 Sequence Detection System (Applied Biosystems, Foster City, CA, USA) using SYBR^®^ Premix DimerEraser^TM^ Kit (TaKaRa, Japan). PCR conditions were: 5 s at 95°C, 30 s at 55°C and 45 s at 72°C, and a total of 45 cycles for real-time PCR. Forward and reverse oligonucleotide primers in the 5’ to 3’ orientation were:

IL-12p40, GGAAGCACGGCAGCAGAATA and AACTTGAGGGAGAAGTAGGAATGG3;
TGF-β, TTGCTTCAGCTCCACAGAGA and TGGTTGTAGAGGGCAAGGAC;
IL-10, CCAAGCCTTATCGGAAATGA and TTTTCACAGGGGAGAAATCG;
FasL, GCAAATAGCCAACCCCAGTACAC and GCCACCTTTCTTATACTTCACTCCAG;
Arginase I, ATCAACACTCCCCTGACAACC and CGCAAGCCAATGTACACGAT;
IDO, CAAGGGCTTCTTCCTCGTCT and GGTCCACAAAGTCACGCATC;

β-actin, ATCCGTAAAGACCTCTATGC and ACACAGAGTACTTGCGCTCA. Relative gene expression levels were determined as described [31]. PCR results were normalized to the expression of the housekeeping gene β-actin. Data shown were representative of six independent experiments. 

### Proliferation assay

Responder cells were CD4^+^ T cells, which were isolated from splenic single cell suspensions (SC) of CIA mice (devoted on Day 49 after the primary immunization) using a CD4^+^ T Cell Isolation Kit (Miltenyi Biotec, Germany). mDCs loaded with CII, were derived from normal D1 mice and induced as described above and used in the proliferation assay as stimulator APCs. A total of 1×10^5^ responding CD4^+^ T cells labeled with 5 μg of carboxyfluorescein succinimidyl ester (CFSE; Invitrogen, Germany). These responder cells were activated following co-culture with 2×10^4^ mDC in 96-well U-bottom culture plates. The allo-tDCs (as suppressor cells) or modified allo-tDCs, which were derived from B6 mice, were then added to the responder cultures at different ratios in a final volume of 200 μl. Four days later, the cells were harvested, stained with propidium iodide (PI, Sigma) and analyzed by flow cytometry. Suppressor activity was measured as the frequency of dividing CFSE^+^ cells in the co-culture and in the presence or absence of allo-tDCs.

### Treatment of CIA mice with allo-tDCs

For *in vivo* experiments, various numbers (10^6^, 10^5^ and 10^4^) of donor allo-tDCs were adoptively transferred via the tail vein into CIA recipient mice (n=10 per group) when onset of CIA was confirmed (the 4th week after the primary immunization). Modified allo-tDCs, such as allo-mtDC, and allo-CII-mtDCs described above, were similarly administrated to recipient mice (n=10). Control mice were treated with PBS alone. Mice were scored for clinical signs of disease from the 4th to 7th week after the primary immunization. Moreover, allo-tDCs (5×10^5^) labeled with CFSE were transferred i.v when the onset of CIA was confirmed. After 7 days, mononuclear cells were isolated from the blood, BM, spleen, liver, lymph node and feet. The MHC molecule of the H-2K^b^ haplotype was determined by flow cytometry.

### Histology

Hind paws were collected and fixed in 4% PFA and decalcified in 15% EDTA and 30% glycerol. Tissue was then dehydrated in a gradient of ethanol solutions, paraffin embedded, sectioned in 5 um specimen slices, mounted on glass slides, and stained with H&E, Safranin O or Toluidine blue. Two independent observers who were blinded to the experimental groups examined the paw sections. Using a four-point scale: normal, 0; inflammatory infiltrates and synovial hyperplasia 1; pannus formation and cartilage erosion, 2; and import cartilage erosion and bone destruction, 3. this global histological score reflected both synovitis (synovial proliferation, inflammatory cell infiltration) and joint destruction (bone and cartilage thickness, irregularity, and presence of erosions) [32].

### ELISA of anti-CII antibodies in serum

Serum was obtained from CIA mice, which were treated with allo-tDC on Days 28, 35, 42 and 49 after the primary immunization and stored at -80°C. Anti-CII antibodies were measured using a standard sandwich ELISA (Chondrex, Redmond, WA, USA) according to the manufacturer’s instructions. 

### Serum cytokine analysis by CBA and ELISA

Serum was obtained from the allo-tDC-treated groups and CIA group on day 49 after the primary immunization, and stored at -80°C until assayed. Levels of IFNγ, TNFα, IL-17, IL-6 and IL-10 were simultaneously determined by the mouse cytokine cytometric bead array (CBA Kit, BD Pharmingen) according to the manufacturer’s instructions. Cytokine bead staining was analyzed by flow cytometry and the data were compiled using CBA software (BD Biosciences). The concentration of TGFβ in the serum was assessed by the mouse TGFβ1 Platinum ELISA kit (eBioscience) according to the manufacturer’s instructions. A total of five samples were analyzed in each group.

### Analysis of Treg/Th17 subsets in CIA mice

The T cells that were isolated from the spleen and inguinal lymph nodes were obtained from the allo-tDC-treated groups and the CIA group on day 49 after the primary immunization. The T cells were incubated with anti-mouse CD4-FITC mAb, fixed, permeabilized, and stained with anti-mouse FoxP3-PE, IL-17-PE or isotype control mAbs (BD Biosciences Pharmingen). 

### Statistical analysis

Results were analyzed by GraphPad Prism 5.0 software (GraphPad Software, San Diego, CA, USA), and data were expressed as the mean ± SEM. Student’s T-test was used to assess statistical significance between two paired groups, and nonparametric tests were used to assess statistical significance among multiple groups. For all tests, an alpha value of *P* <0.05 was considered statistically significant.

## Supporting Information

Figure S1 **Phenotype of tDCs derived from B6 mice.** tDCs were induced in vitro as described in the Materials and Methods section, following which non-adherent cells were harvested. Non-adherent cells were stained with CD11c, Foxp3, CD11b and Gr1 or isotype-matched mAbs and the expression of those markers analyzed by FACS flow cytometry.
(TIF)

Figure S2 **Allo-tDCs could not induce the proliferation of the CD4^+^ and CD8^+^ T cells.** In the Proliferation assays, CD4^+^T cell (responders) were isolated from CIA mice (H-2Kq) SC, which were then stained with CFSE, and stimulated with CII-loaded mDCs (stimulators), which were derived from bone marrow precursors of normal D1 mice. tDCs/mtDCs derived from D1 and B6 mice were added to the proliferation assay at an S:R ratio of 1:100, respectively. Additionally, CD4/CD8T cells alone were defined as the negative control and CD4/CD8T cells stimulated by mDC (D1) were defined as the positive control. After co-culture for 4 d, cells were harvested and analyzed by FACS. Progressive dilution of CFSE was used as a measure of cell proliferation.
(TIF)

Figure S3 **The FACS data of the H-2K^b+^ cells in CIA mice without adoptive transfer of allo-tDCs.** As the negative control of FACS, the samples of blood, BM, spleen, liver, lymph node and feet from the CIA mice without transfer of allo-tDCs were collected. And the Mononuclear cells from these samples were isolated and stained H-2K^b^, which is specific MHC molecule of B6 mice. And H-2K^b+^ cells were determined by flow cytometry and the representative data were shown.
(TIF)

Figure S4 **Therapeutically optimal doses of autologous tDCs promoted an antigen-specific anti-arthritic activity in CIA mice.** (**A**) Three different doses of tDCs (ranging from 5×10^6^ to 5×10^4^ cells) derived from D1 mice were adoptively transferred following the onset of experimentally induced CIA. (**B**) D1-tDCs were stimulated by LPS and pulsed with either CII peptide, OVA (as an irrelevant antigen), or the DC were left untreated, and adoptively transferred into CIA mice at the same density (5×10^5^/animal). Mice were scored for clinical signs of arthritis in the limb joints by macroscopic examination three times a week. Limb joint arthritis was assessed by an established scoring system. Arthritic score and incidence following adoptive transfer of different doses of allo-tDC in each group (n=5) during the observation period are shown. *P <0.05 as compared with CIA mice by unpaired *t*-test analysis.
(TIF)
