# Supplementary figures and images for: A Mouse Model of Adoptive Immunotherapeutic Targeting of Autoimmune Arthritis Using Allo-Tolerogenic Dendritic Cells
Source: PLoS One. 2013 Oct 24;8(10):e77729. doi: 10.1371/journal.pone.0077729 (PMC3812020; doi:10.1371/journal.pone.0077729)

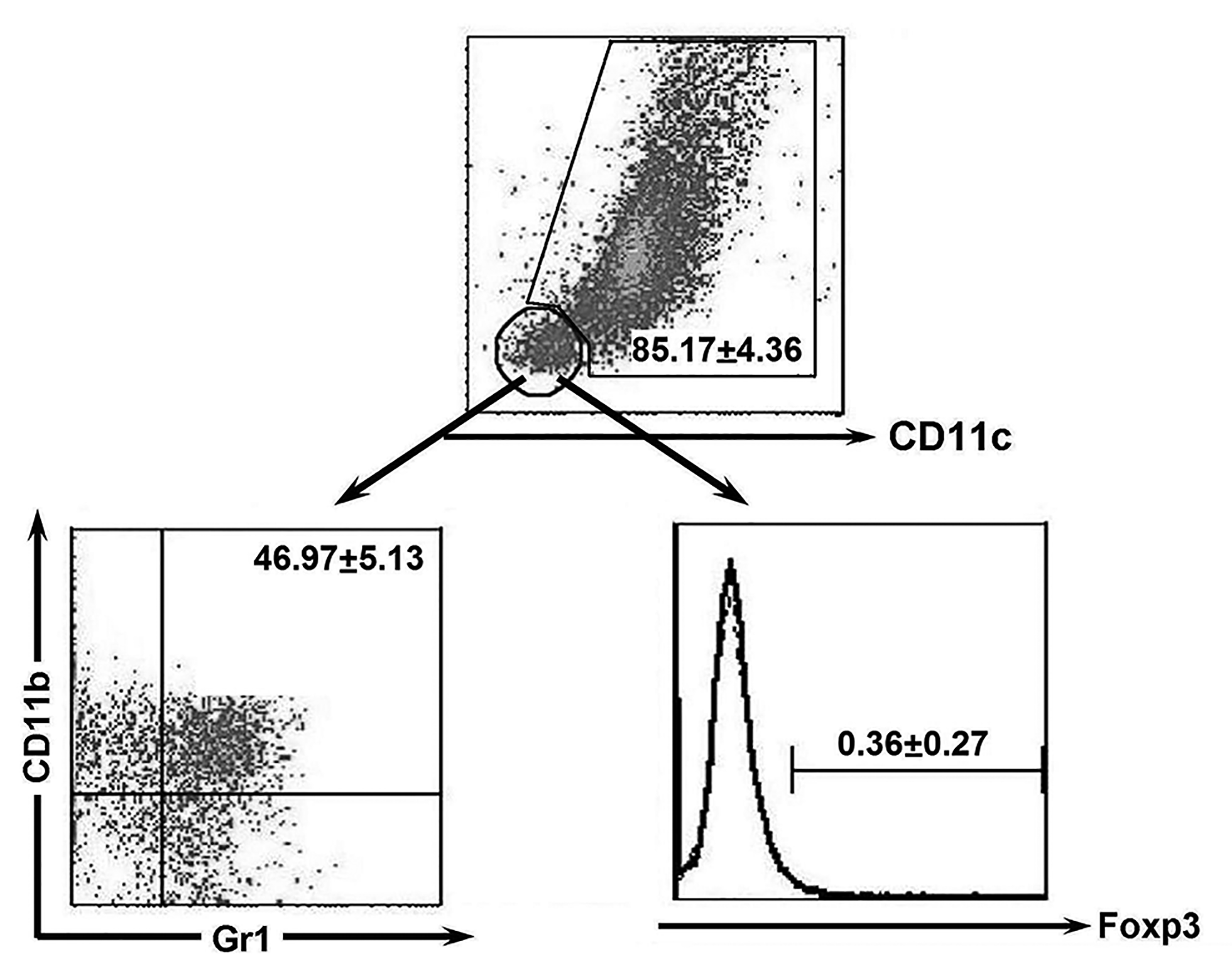

Supplement: Figure S1 — Phenotype of tDCs derived from B6 mice. tDCs were induced in vitro as described in the Materials and Methods section, following which non-adherent cells were harvested. Non-adherent cells were stained with CD11c, Foxp3, CD11b and Gr1 or isotype-matched mAbs and the expression of those markers analyzed by FACS flow cytometry. (TIF) [file pone.0077729.s001.tif]

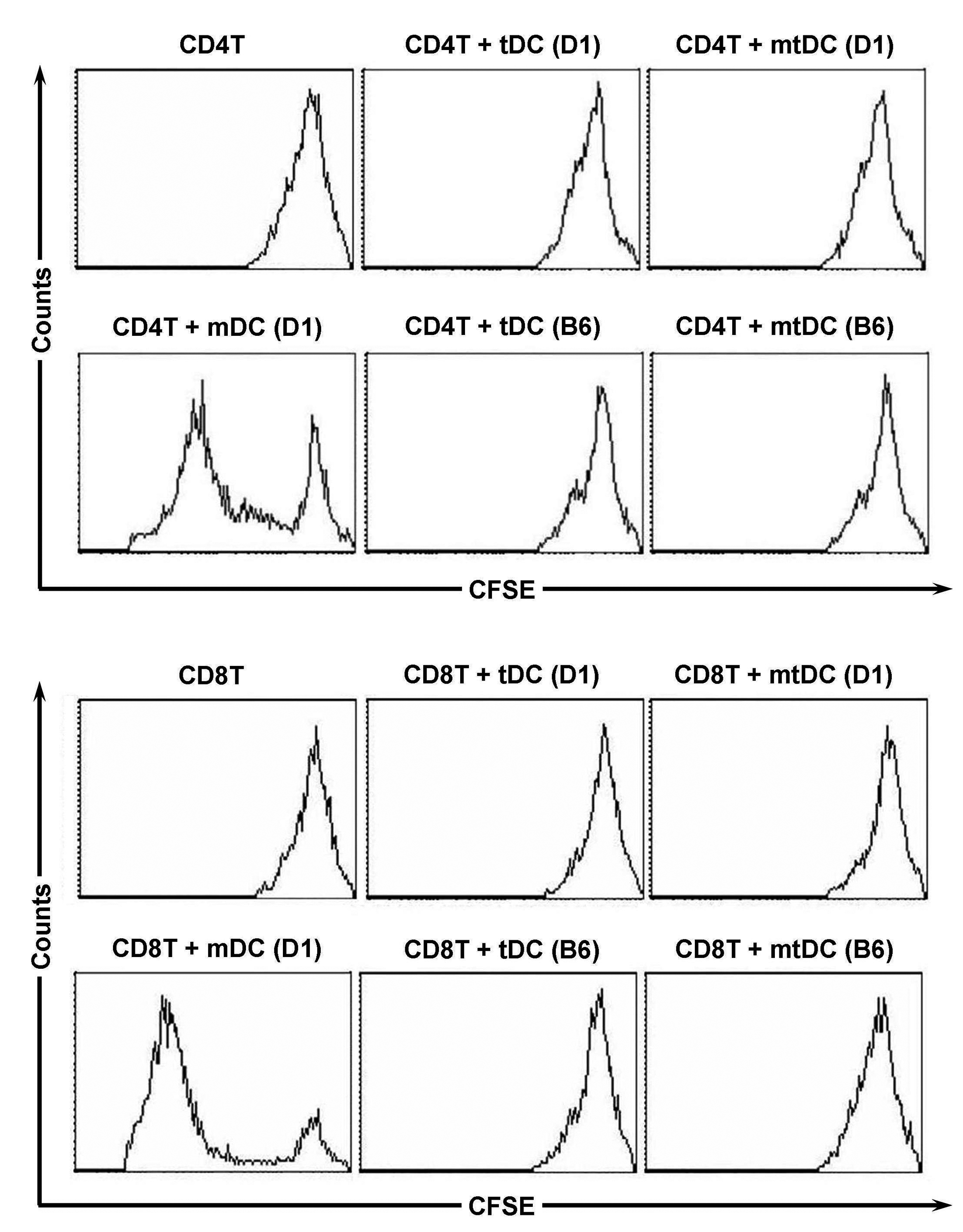

Supplement: Figure S2 — Allo-tDCs could not induce the proliferation of the CD4+ and CD8+ T cells. In the Proliferation assays, CD4+T cell (responders) were isolated from CIA mice (H-2Kq) SC, which were then stained with CFSE, and stimulated with CII-loaded mDCs (stimulators), which were derived from bone marrow precursors of normal D1 mice. tDCs/mtDCs derived from D1 and B6 mice were added to the proliferation assay at an S:R ratio of 1:100, respectively. Additionally, CD4/CD8T cells alone were defined as the negative control and CD4/CD8T cells stimulated by mDC (D1) were defined as the positive control. After co-culture for 4 d, cells were harvested and analyzed by FACS. Progressive dilution of CFSE was used as a measure of cell proliferation. (TIF) [file pone.0077729.s002.tif]

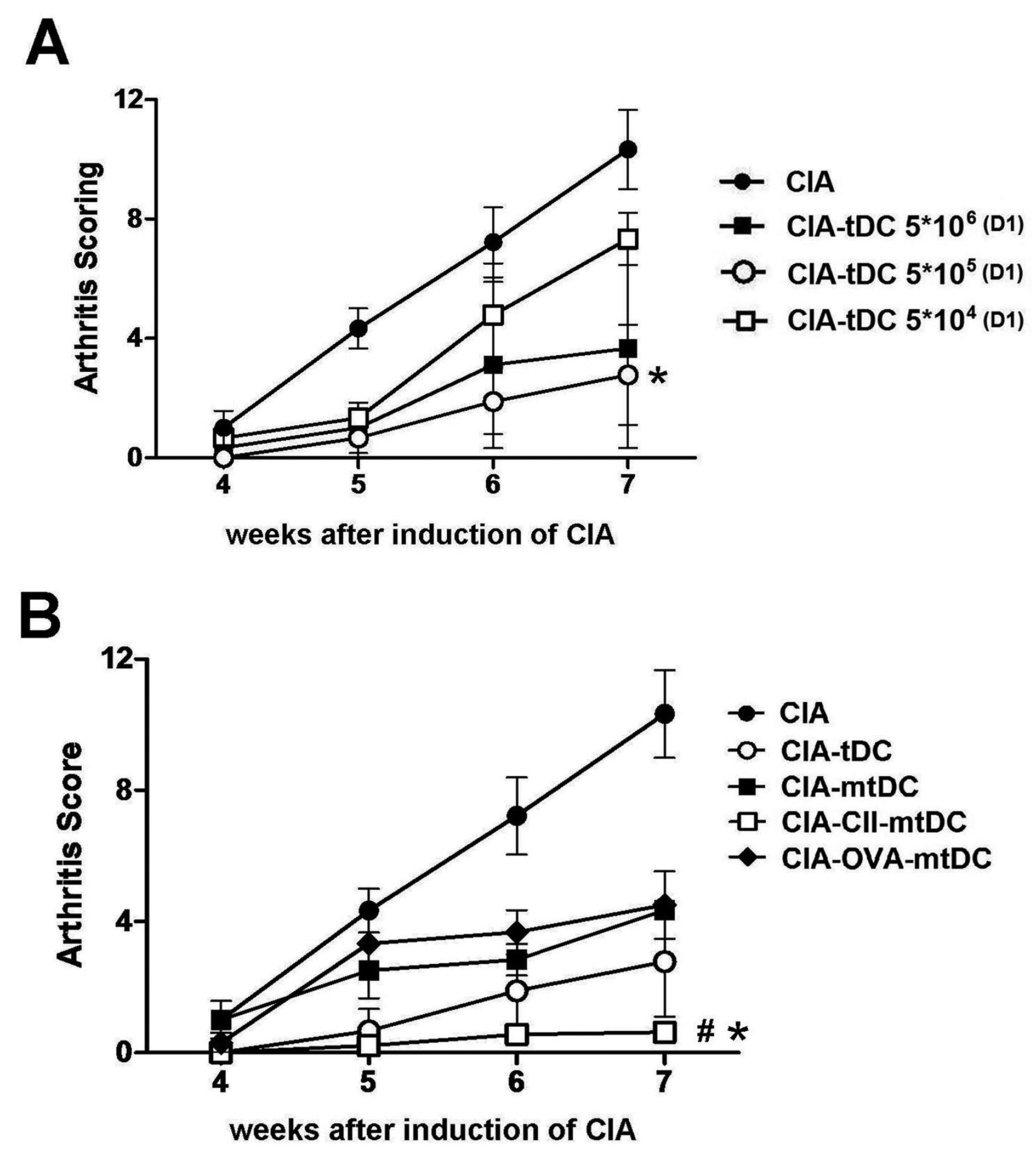

Supplement: Figure S4 — Therapeutically optimal doses of autologous tDCs promoted an antigen-specific anti-arthritic activity in CIA mice. (A) Three different doses of tDCs (ranging from 5×10^6^ to 5×10^4^ cells) derived from D1 mice were adoptively transferred following the onset of experimentally induced CIA. (B) D1-tDCs were stimulated by LPS and pulsed with either CII peptide, OVA (as an irrelevant antigen), or the DC were left untreated, and adoptively transferred into CIA mice at the same density (5×10^5^/animal). Mice were scored for clinical signs of arthritis in the limb joints by macroscopic examination three times a week. Limb joint arthritis was assessed by an established scoring system. Arthritic score and incidence following adoptive transfer of different doses of allo-tDC in each group (n=5) during the observation period are shown. *P <0.05 as compared with CIA mice by unpaired t-test analysis. (TIF) [file pone.0077729.s004.tif]
